# Convergence of Mortality Rates among Patients on Antiretroviral Therapy in South Africa and North America

**DOI:** 10.1371/journal.pmed.1001719

**Published:** 2014-09-09

**Authors:** Agnes Binagwaho, Cameron T. Nutt, Placidie Mugwaneza, Claire M. Wagner, Sabin Nsanzimana

**Affiliations:** 1 Ministry of Health of Rwanda, Kigali, Rwanda; 2 Department of Global Health and Social Medicine, Harvard Medical School, Boston, Massachusetts, United States of America; 3 Geisel School of Medicine at Dartmouth, Hanover, New Hampshire, United States of America; 4 Partners In Health, Boston, Massachusetts, United States of America; 5 HIV and Other Sexually Transmitted Infections Division, Rwanda Biomedical Center, Kigali, Rwanda; 6 Center for Global Cancer Medicine, Dana Farber Cancer Institute, Boston, Massachusetts, United States of America

## Abstract

Agnes Binagwaho and colleagues explore the narrowing gap between South African and North American cohorts in survival on HIV treatment, described in the study by Andrew Boulle and colleagues.

*Please see later in the article for the Editors' Summary*

**Linked Research Article**

This Perspective discusses the following new study published in *PLOS Medicine*:

Boulle A, Schomaker M, May MT, Hogg RS, Shepherd BE, et al. (2014) Mortality in Patients with HIV-1 Infection Starting Antiretroviral Therapy in South Africa, Europe, or North America: A Collaborative Analysis of Prospective Studies. PLoS Med 11(9): e1001718. doi:10.1371/journal.pmed.1001718

Analyzing survival in HIV treatment cohorts, Andrew Boulle and colleagues find mortality rates in South Africa comparable to or better than those in North America by 4 years after starting antiretroviral therapy.

In the early 2000s, the pages of medical journals were filled with (often rancorous) debates about the feasibility and prudence of expanding access to antiretroviral therapy (ART) in sub-Saharan Africa, where HIV disease had become the leading cause of death among young adults. Despite a paucity of supporting data, some public health experts warned of “antiretroviral anarchy” in Africa and the resulting spread of poly-resistant strains of HIV to wealthy countries [1],[2],[3].

Shortly after HIV treatment scale-up began in earnest across the continent, however, adherence to ART in Africa was found to be significantly higher than in North America [4]. As more patients initiated therapy, it was thus expected that the wide outcome gaps between the two settings would narrow.

In a landmark analysis of data from 67,354 patients in this week's issue of *PLOS Medicine*, Andrew Boulle and colleagues report that all-cause mortality rates among South African patients on ART for longer than 24 months have fallen to levels equal to or below those registered in comparable North American cohorts [5]. Among patients with timely and sustained access to care and for whom data was available, the same proportion as in Europe (89.5%) achieved virological suppression to below 400 copies/ml after 6 months on treatment in South Africa, compared to 73.4% in North America [5].

With more than 2.5 million South Africans on effective treatment (increasingly financed by the national government), the country is now beginning to rein in the world's largest HIV epidemic [6]. Over the same period as Boulle and colleagues' study, the scale-up of ART in rural Kwa-Zulu Natal was associated with an overall gain of 11.3 years of adult life expectancy between 2003 and 2011 [7].

The study also highlights the crucial role of population-based registries in identifying and redressing disparities. More than half of the deaths included in the South African analysis were missed by cohort studies but captured by the national vital registry [5]. In settings with weak or absent registries, clinic-based efforts to ascertain long-term patient outcomes may underestimate mortality.

In line with earlier comparative analyses [8], Boulle and colleagues' study shows that early mortality (within the first 12 months of therapy) remains very high in South Africa, at 9.7%—more than double that in North America (4.6%) and nearly five times that in Europe (2.0%) [5]. While poor long-term outcomes in North America appear to be linked to poor retention in care among marginalized groups, suboptimal adherence, and the impact of highly prevalent co-infections such as chronic viral hepatitis C, the most important driver of mortality in South Africa was delayed access to care: half of South African participants had a CD4 count below 100 cells/µl when they initiated treatment [5].

One in seven patients in the South African studies was lost to follow-up [5]. As noted, linking such patients with national death registries is an important task for researchers and epidemiologists, but closing remaining equity gaps will require linking these patients back to care—keeping them in the cohort and off of the registry. North America and South Africa each had a cumulative incidence of mortality that exceeded Europe's by more than 300% in the study; vulnerable patients continue to fall through the cracks.

Barriers related to poverty, stigma, and race exist along each step of the care continuum for HIV [9]. For example, the advent of highly effective direct-acting antiviral regimens could open the door to substantial improvements in long-term survival in the United States among patients co-infected with HIV and viral hepatitis C. Realizing such gains, however, would require an effective delivery strategy that guarantees access and assists patients in overcoming major barriers to treatment adherence and retention in care [10]. While challenges related to the delivery of care vary widely across contexts, it is possible that certain lessons—especially pertaining to community-based care—learned in rural South Africa or central Haiti might offer a promising way forward for improving treatment outcomes for HIV and other chronic diseases in North America [11].

Rigorous ethnographic studies have revealed structural barriers, such as food insecurity and high transportation costs, as major contributors to loss to follow-up among poor patients, regardless of where they live [10],[12]. As we have noted elsewhere, when taking average family incomes into account, a monthly round-trip bus fare to refill an ART prescription can pose a similar burden in rural Africa as a monthly business class airplane ticket from Los Angeles to Boston in the US [13]. Sustained reductions in mortality will require investing in approaches to strengthening health systems that support patients in overcoming these obstacles, and that harness the social capital shown to facilitate adherence [14].

And what of the many hundreds of thousands of would-be patients who never begin therapy at all? Flat-lined global resources have hamstrung the AIDS response in many settings around the world, and reports of waiting lists for ART have grown increasingly common in Africa in recent years [15].

Disparities of access and outcome that are both regional and intensely local in nature warrant a substantial increase in attention in each of the settings studied. In the US, residents of the District of Columbia are 12 times more likely to die of HIV than those in the neighboring state of Virginia [16]. Deaths due to HIV disease have risen 15-fold since 2000 in Eastern Europe; the AIDS-related mortality rate in Ukraine now exceeds that in Ethiopia and Burkina Faso [17]. In Africa, 27 nations have less than 40% coverage for ART under the latest World Health Organization guidelines [6].

When access is assured and sustained, it is now clear that excellent clinical outcomes are possible, including (or, perhaps, especially) in rural regions of Africa. Far from wishful thinking, the notion of a “grand convergence” in global health through the narrowing of disparities across continents and income groups is both a concrete possibility and a moral imperative, achievable with accelerated commitments and committed partnerships in all settings [18].
